# Supplementary material for: Reconciling Mining with the Conservation of Cave Biodiversity: A Quantitative Baseline to Help Establish Conservation Priorities
Source: PLoS One. 2016 Dec 20;11(12):e0168348. doi: 10.1371/journal.pone.0168348 (PMC5173368; doi:10.1371/journal.pone.0168348)
Supplement: S1 Dataset — (ZIP) [file pone.0168348.s002.zip › Taxa/Serra Norte/SN_2013/N4E/Lista N4E-08.pdf]

CAVIDADE N4E-0008

Classe	Ordem	Fam/Outros	Gên/Outros	Espécie	S-U	U-S	Zon.
Annelida	Oligochaeta			jovem		1	E
Arachnida	Acari	(Oribatida)		jovem	X		
Arachnida	Acari	(Parasitengonina)		sp.2		X	P
Arachnida	Acari	(Parasitengonina)		sp.4		X	P
Arachnida	Acari	(Uropodina)		sp.1		X	P
Arachnida	Acari	Cunaxidae		sp.1		X	P
Arachnida	Acari	Labidostommatidae		sp.1		X	E/P
Arachnida	Acari	Laelapidae		sp.3		X	E/P
Arachnida	Acari	Mesostigmata		jovem	X		
Arachnida	Acari	Prostigmata		jovem	X		
Arachnida	Acari	Trombiculidae		sp.39	X	X	E
Arachnida	Araneae	Araneidae	*Alpaida*	sp.1	X		
Arachnida	Araneae	Barychelidae		jovem		4	E
Arachnida	Araneae	Caponiidae		sp.1		X	P
Arachnida	Araneae	Corinnidae		jovem		4	E/P
Arachnida	Araneae	Ctenidae		jovem	1	1	E
Arachnida	Araneae	Drymusidae	*Drymusa*	*spelunca*		X	E
Arachnida	Araneae	Filistatidae		jovem		X	E
Arachnida	Araneae	Mysmenidae	*Microdipoena*	sp.1		X	E
Arachnida	Araneae	Ochyroceratidae	*Ochyrocera*	sp.3	X	X	E/P
Arachnida	Araneae	Ochyroceratidae	*Speocera*	sp.1	X	X	E/P
Arachnida	Araneae	Pholcidae	*Leptopholcus*	sp.1		X	P
Arachnida	Araneae	Pholcidae	*Mesabolivar*	sp.1		X	E
Arachnida	Araneae	Pholcidae	Ninetinae	sp.1		X	E
Arachnida	Araneae	Pholcidae		jovem	X	X	E
Arachnida	Araneae	Salticidae		sp.1		X	E
Arachnida	Araneae	Segestriidae	*Ariadna*	sp.	X		
Arachnida	Araneae	Tetragnathidae		jovem		X	E
Arachnida	Araneae	Theraphosidae	aff. *Holothele*	sp.1	X	1	E
Arachnida	Araneae	Theridiidae	*Theridion*	sp.	X		
Arachnida	Araneae	Theridiidae	*Thymoites*	sp.	X		
Arachnida	Araneae	Theridiosomatidae	*Plato*	sp.1	X	X	E/P
Arachnida	Araneae	Uloboridae	*Uloborus*	sp.1		X	E
Arachnida	Opiliones	Escadabiidae		sp.2	X	X	E/P
Arachnida	Opiliones	Sclerosomatidae	Gagrellinae	sp.	X		
Arachnida	Opiliones	Sclerosomatidae	*Prionostema*	sp.1		1	E
Arachnida	Opiliones	Stygnidae	*Protimesius*	*gracilis*		1	E
Arachnida	Palpigradi	Eukoeneniidae	*Allokoenenia*	sp.1	X	X	P
Arachnida	Pseudoscorpiones	Bochicidae		sp.1		X	P
Arachnida	Pseudoscorpiones	Chernetidae	*Spelaeochernes*	sp.1	X	X	E
Chilopoda	Geophilomorpha	Geophilidae		sp.1		1	E
Chilopoda	Scolopendromorpha	Scolopocryptopidae	*Tidops*	sp.1		1	E
Chilopoda	Scolopendromorpha	Scolopocryptopidae		sp.4		1	E
Chilopoda	Scutigeromorpha	Pselliodidae	*Sphendononema*	*guildingii*	X		
Diplopoda	Polydesmida	Chelodesmidae		sp.3		1	P
Diplopoda	Polydesmida	Fuhrmannodesmidae		jovem		X	P
Diplopoda	Polydesmida	Pyrgodesmidae		sp.1		X	P
Entognatha	Collembola	Entomobryidae		sp.6		X	E
Entognatha	Collembola	Isotomidae		sp.1		X	E/P
Entognatha	Collembola	Paronellidae		sp.1		X	E
Entognatha	Collembola	Paronellidae		sp.4		X	E/P
Entognatha	Collembola	Sminthuroidea		sp.2	X	X	E/P
Entognatha	Diplura	Campodeidae		sp.1	X	X	E
Entognatha	Diplura	Japygidae		sp.1		X	E
Entognatha	Diplura	Projapygidae		sp.1	X	X	E

Insecta	Blattodea	Polyphagidae		jovem		1	E/P
Insecta	Coleoptera	Carabidae	*Coarazuphium*	sp.n.1		X	P
Insecta	Coleoptera	Carabidae	*Coarazuphium*	sp.n.2	X		P
Insecta	Coleoptera	Curculionidae	Scolytinae	sp.4		X	E
Insecta	Coleoptera	Curculionidae	Scolytinae	sp.5		X	E
Insecta	Coleoptera	Elateridae		jovem	X		
Insecta	Coleoptera	Lycidae		sp.1	X		
Insecta	Coleoptera	Scydmaenidae		sp.3		X	P
Insecta	Coleoptera	Staphylinidae	Pselaphinae	sp.34		X	E
Insecta	Diptera	Aulacigastridae		sp.		X	E
Insecta	Diptera	Culicidae	*Culex*	sp.	X	X	P
Insecta	Diptera	Curtonotidae		sp.	X		
Insecta	Diptera	Dixidae		sp.	X		
Insecta	Diptera	Drosophilidae	*Drosophila*	sp.	X		
Insecta	Diptera	Phoridae	Metopininae	sp.	X	X	E
Insecta	Diptera	Psychodidae	Phlebotominae	sp.		X	E/P
Insecta	Diptera	Psychodidae	*Pintomyia*	*gruta*	X		
Insecta	Diptera	Psychodidae	*Sciopemyia*	*sordellii*	X		
Insecta	Diptera	Sciaridae		sp.		X	P
Insecta	Diptera	Tipulidae	Tipulinae	sp.		X	E
Insecta	Heteroptera	Cydnidae		sp.	X	X	P
Insecta	Heteroptera	Nabidae	*Arachnocoris*	jovem		X	E
Insecta	Heteroptera	Reduviidae	Emesinae	sp.		2	E
Insecta	Heteroptera	Reduviidae	*Zelurus*	sp.	X	2	E
Insecta	Homoptera	Cixiidae		jovem		X	E
Insecta	Hymenoptera	Diapriidae		sp.2		X	P
Insecta	Hymenoptera	Evaniidae	*Evania*	sp.	X		
Insecta	Hymenoptera	Formicidae	*Apterostigma*	sp.	X		
Insecta	Hymenoptera	Formicidae	*Brachymyrmex*	sp.	X		
Insecta	Hymenoptera	Formicidae	*Camponotus*	sp.	X		
Insecta	Hymenoptera	Formicidae	*Camponotus*	sp.1		X	E/P
Insecta	Hymenoptera	Formicidae	*Camponotus*	sp.2		X	E
Insecta	Hymenoptera	Formicidae	*Cephalotes*	sp.1		X	P
Insecta	Hymenoptera	Formicidae	*Dolichoderus*	aff. *bispinosus*	X	X	E
Insecta	Hymenoptera	Formicidae	*Gnamptogenys*	sp.1		X	E
Insecta	Hymenoptera	Formicidae	*Gnamptogenys*	sp.3		X	E
Insecta	Hymenoptera	Formicidae	*Hypoponera*	sp.1		X	E/P
Insecta	Hymenoptera	Formicidae	*Leptogenys*	sp.1	X		
Insecta	Hymenoptera	Formicidae	*Pachycondyla*	*constricta*	X		
Insecta	Hymenoptera	Formicidae	*Pachycondyla*	sp.1		X	E
Insecta	Hymenoptera	Formicidae	*Paratrechina*	sp.	X		
Insecta	Hymenoptera	Formicidae	*Pheidole*	sp.1		X	E
Insecta	Hymenoptera	Formicidae	*Solenopsis*	sp.1		X	E/P
Insecta	Hymenoptera	Scelionidae		sp.	X		
Insecta	Isoptera	Termitidae	*Labiotermes*	sp.		X	E
Insecta	Isoptera	Termitidae	*Nasutitermes*	sp.		X	E
Insecta	Isoptera	Termitidae		sp.	X		
Insecta	Lepidoptera	Tineoidea		sp.	X		
Insecta	Lepidoptera	Tineoidea		sp.1		X	P
Insecta	Lepidoptera	Tineoidea		sp.2		X	E
Insecta	Neuroptera	Myrmeleontidae		jovem		X	E
Insecta	Orthoptera	Phalangopsidae	*Phalangopsis*	sp.	X	2	P
Insecta	Plecoptera			sp.	X		
Insecta	Psocoptera	Epipsocidae	*Epipsocus*	sp.1		X	E
Insecta	Psocoptera	Psocomorpha		jovem	X		
Insecta	Psocoptera	Psyllipsocidae		jovem		X	E
Insecta	Psocoptera	Ptiloneuridae	*Triplocania*	sp.3		X	E
Insecta	Thysanura	Nicoletiidae		sp.1		X	E

Gastropoda	Pulmonata	Bulimulidae	*Naesiotus*	sp.		X	E/P
Gastropoda	Pulmonata	Subulinidae	*Lamellaxis*	sp.	X		
Gastropoda	Pulmonata	Systrophiidae	*Happia*	sp.	X	X	E/P
Malacostraca	Isopoda	Armadillidae		sp.1		X	E
Malacostraca	Isopoda	Philosciidae		sp.1		X	E/P
Malacostraca	Isopoda	Scleropactidae	*Circoniscus*	*buckupi*	X		
Nematoda				sp.1		X	E
Pauropoda	Tetramerocerata			sp.		X	E
Symphyla		Scutigerellidae		sp.		X	E
Amphibia	Anura			sp.		4	E
Mammalia	Chiroptera	Phyllostomidae	*Carollia*	*perspicillata*		11	E/P
Mammalia	Chiroptera	Phyllostomidae	*Glossophaga*	*soricina*		20	E/P
Mammalia	Chiroptera			sp.	50		
